# Topographical distribution of neurovascular canals and foramens in the mandible: avoiding complications resulting from their injury during oral surgical procedures

**DOI:** 10.1016/j.heliyon.2018.e00812

**Published:** 2018-09-21

**Authors:** Ayumi Moro, Shigehiro Abe, Naoko Yokomizo, Yutaka Kobayashi, Takashi Ono, Toshiaki Takeda

**Affiliations:** a Department of Dentistry and Oral Surgery, Tokyo Metropolitan Hiroo Hospital, 2-34-10 Ebisu, Shibuya-ku, Tokyo 150-0013, Japan; b Department of Radiology, Tokyo Metropolitan Hiroo Hospital, 2-34-10 Ebisu, Shibuya-ku, Tokyo 150-0013, Japan

**Keywords:** Surgery, Neurology, Anatomy

## Abstract

**Purpose:**

Certain oral surgical procedures can injure neurovascular canals and foramens in the mandible. Hence, before performing surgical procedures, it is important to assess the distribution of the bifid mandibular canal (BMC), accessory mental foramen (AMF), medial lingual canal (MLC), lateral lingual canal (LLC), buccal foramen (BF), and lingual alveolar canal (LAC). This study aimed to assess the distribution of different types of canals and foramens. Furthermore, we investigated the limitations associated with finding these structures in panoramic images.

**Methods:**

Fifty-eight patients who had undergone panoramic radiography and computed tomography (CT) scans at our hospital were randomly selected for this study. Imaging data obtained from these patients were retrospectively reviewed.

**Results:**

We found that the occurrence of BMC was 60.3%, AMF was 6.9%, MLC was 98.2%, LLC was 75.9%, BF was 43.1%, and LAC was 98.3%. Edge-contrasted inverted panoramic images revealed BMCs in 21.7% and AMFs in 25%; however, most of these canals could not be detected. In the panoramic images, the average diameter of the BMC was significantly different between the detected group and not detected group. The number of canals and foramens in the anterior region to the molar region decreased on the buccal and lingual sides, and most BMCs were in the retromolar to the ramus region.

**Conclusion:**

Our results indicated different distributions and occurrence rates of each type of neurovascular canal and foramens.

## 1 Introduction

Damage to neurovascular canals and foramens in the mandible during oral surgical procedures causes many complications, the most serious of which are sensory disturbances and bleeding [1, 2, 3, 4, 5, 6].

The neurovascular canals and foramens consist of the bifid mandibular canal (BMC) [1, 7, 8, 9, 10, 11, 12, 13, 14, 15, 16, 17], accessory mental foramen (AMF) [18], medial lingual canal (MLC) [19], lateral lingual canal (LLC) [19], buccal foramen (BF) [20], and lingual alveolar canal (LAC) [21]. Several researchers have used panoramic images and computed tomography (CT) images to classify the BMC, based on its anatomical location and configuration [8, 12, 17, 22]. The detection rate of the BMC is better with CT images (10.2%–64.8%) than with panoramic images (0.08%–8.3%) [1, 5, 8, 10]. The detection rate of the AMF ranges from <5% to approximately 30%, and it is rarely detected on panoramic images [18]. Panoramic images are often used as a preoperative diagnostic tool in oral surgical procedures; however, the data indicate that the full extent of the BMC and AMF has not been detected. Previously reported detection rates for the MLC, LLC, BF, and LAC are 97.0%, 99.0%, 44%, and 95.5%, respectively [19, 20, 21]. Previous studies report the expression rates and diameters of each type of canal and foramen; however, not all types occur in the same patient, so it is very important to evaluate their distribution in the mandible. Furthermore, the presence of these structures in panoramic images is important for screening.

The aim of this study was to assess the distribution of all types of neurovascular canals and foramens in the mandible within the same patient. A further aim was to obtain findings to improve the discovery rate in panoramic images, based on the evidence of these canals and foramens in CT images. These results may contribute to more accurate diagnoses and avoid complications during oral surgical techniques.

## 2 Materials and methods

### 2.1 Study subjects

The institutional review board of Tokyo Metropolitan Hiroo Hospital approved the study design (review approval no. 2016-17). This study investigated 104 sides from 58 randomly selected patients who had undergone panoramic radiography and CT scans at our hospital from April 2015 to March 2016 [23]. Patients with cystic lesions, odontogenic tumors, and other lesions in the hemimandibles were excluded.

### 2.2 Imaging system

An Aquilion TSX-101-H scanner (Toshiba, Tochigi, Japan) was used to acquire multislice CT (MSCT) images with the following parameters: detector coverage of 100–120 mm, 512 × 512 matrix with a 16-cm field of view, section thickness and interval of 0.5 mm. The exposure volume was set at 120 kV (tube voltage) and 80 mA (tube current) with a rotation time of 1.0 second. The dental images (panoramic and cross-sectional images) using dedicated dental CT software were reconstructed with 0.5-mm slices. The voxel size of the dental images was 0.3125 × 0.3125 × 0.5 mm. The coronal, axial, and sagittal images were also reconstructed with 2.0-mm slice thickness. Panoramic imaging was performed using the Auto 1000EX scanner (Asahi Roentgen Industry Co., Ltd., Kyoto, Japan).

### 2.3 Neurovascular canal and foramen classification

Based on continuous MSCT images, the BMC were classified by using a modified version of Naitoh's classification system [8, 15]. Furthermore, the AMF, MLC, LLC, BF, and LAC were detected. For the MLC, 56 patients were evaluated with CT images; two patients were excluded because the inferior border of the mandibles were not obtained.

**Fig. 1:**
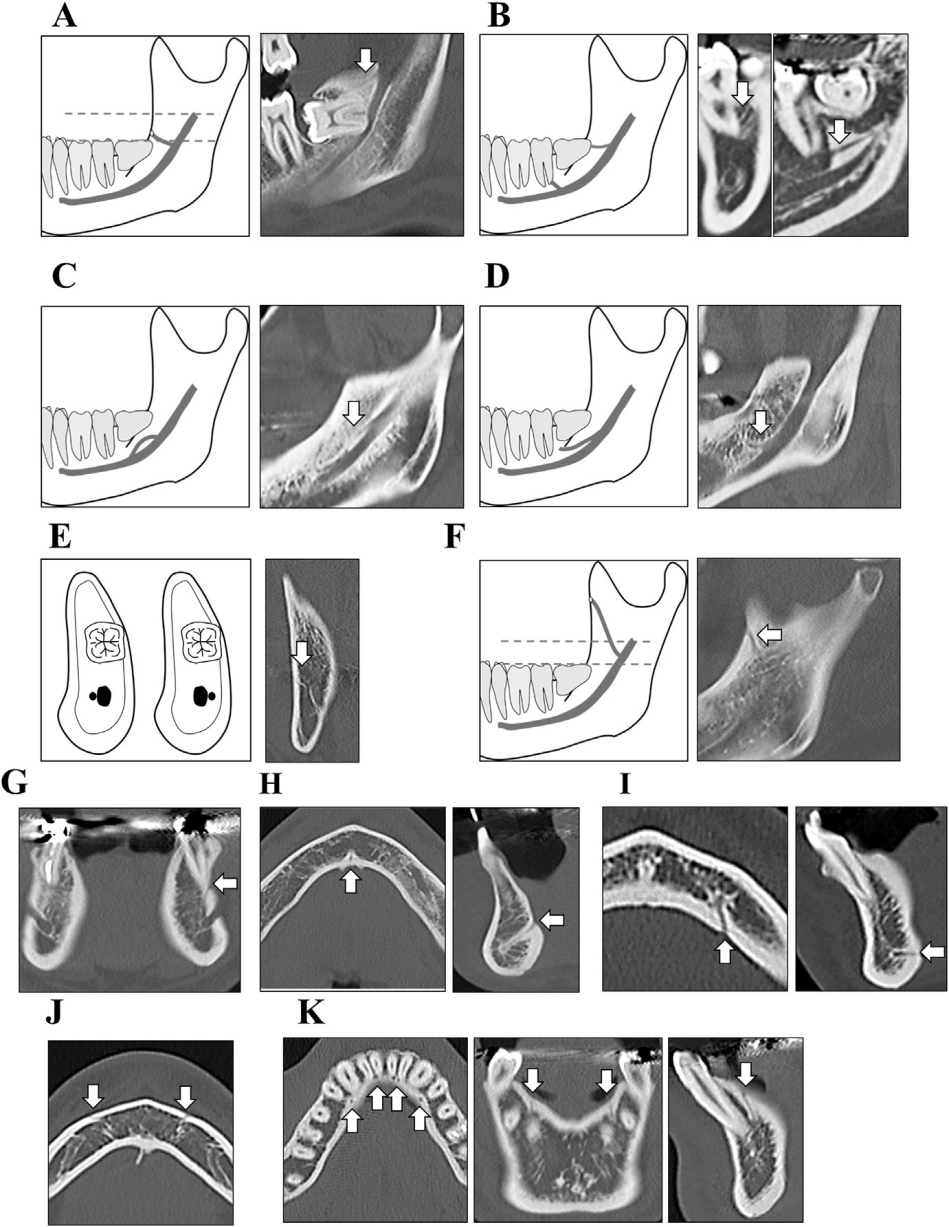
Diagrams and computed tomography images of the configuration of bone canals and foramens. The bifid mandibular canal is classified into five types: (A) retromolar canal; (B) dental canal; (C and D) forward canal, which is classified as “with confluence” (C) or without confluence (D); (E) buccolingual canal; and (F) ramus canal. Other bone canals are (G) the accessory mental foramen, (H) median lingual canal, (I) lateral lingual canal, (J) buccal foramen, and (K) lingual alveolar canal.

**Fig. 2:**
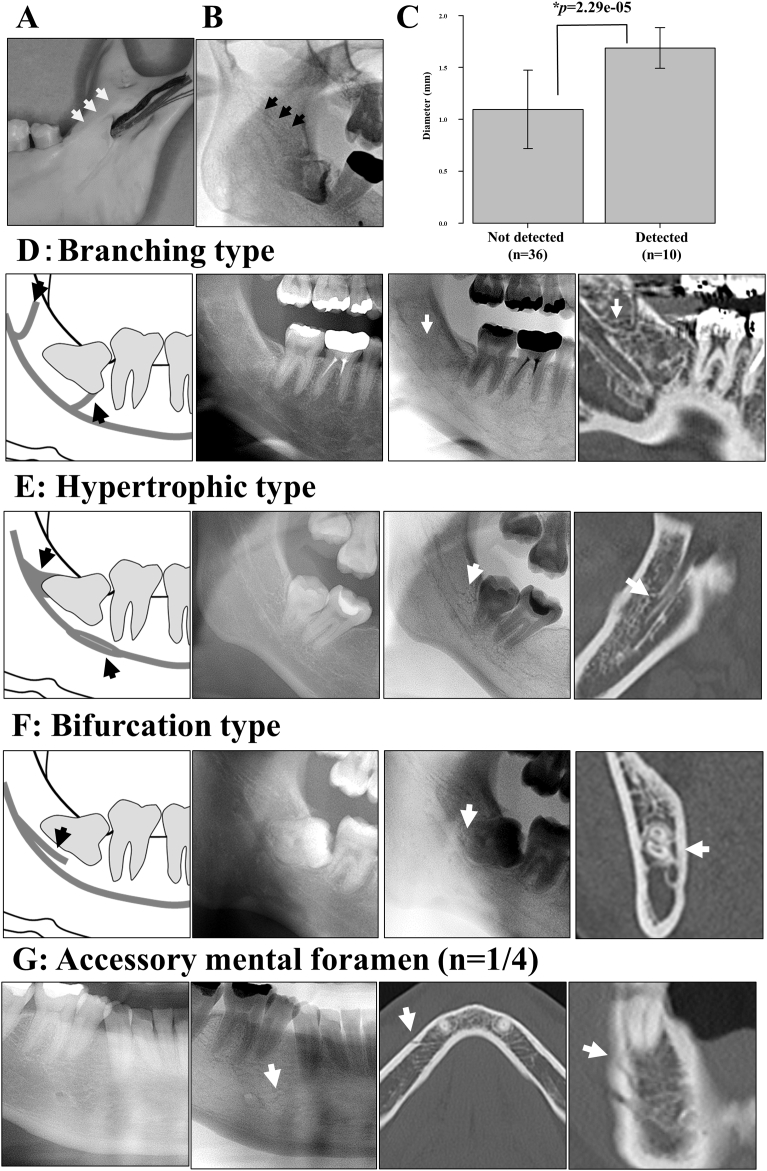
Bifid mandibular canals and accessory mental foramens on edge-contrasted inverted panoramic images. (A) The inner surface of ramus of mandibular bone model. (B) The failure shading of inner surface of mandibular ramus. (C) The mean diameter of bifid mandibular canal between the detected and not detected group. (D–F) There are three types of detectable canals: the branching type (D), the hypertrophic type (E), and the bifurcation type (F). (G) The accessory mental foramen is detected on the panoramic image. *P < 0.01.

**Fig. 3:**
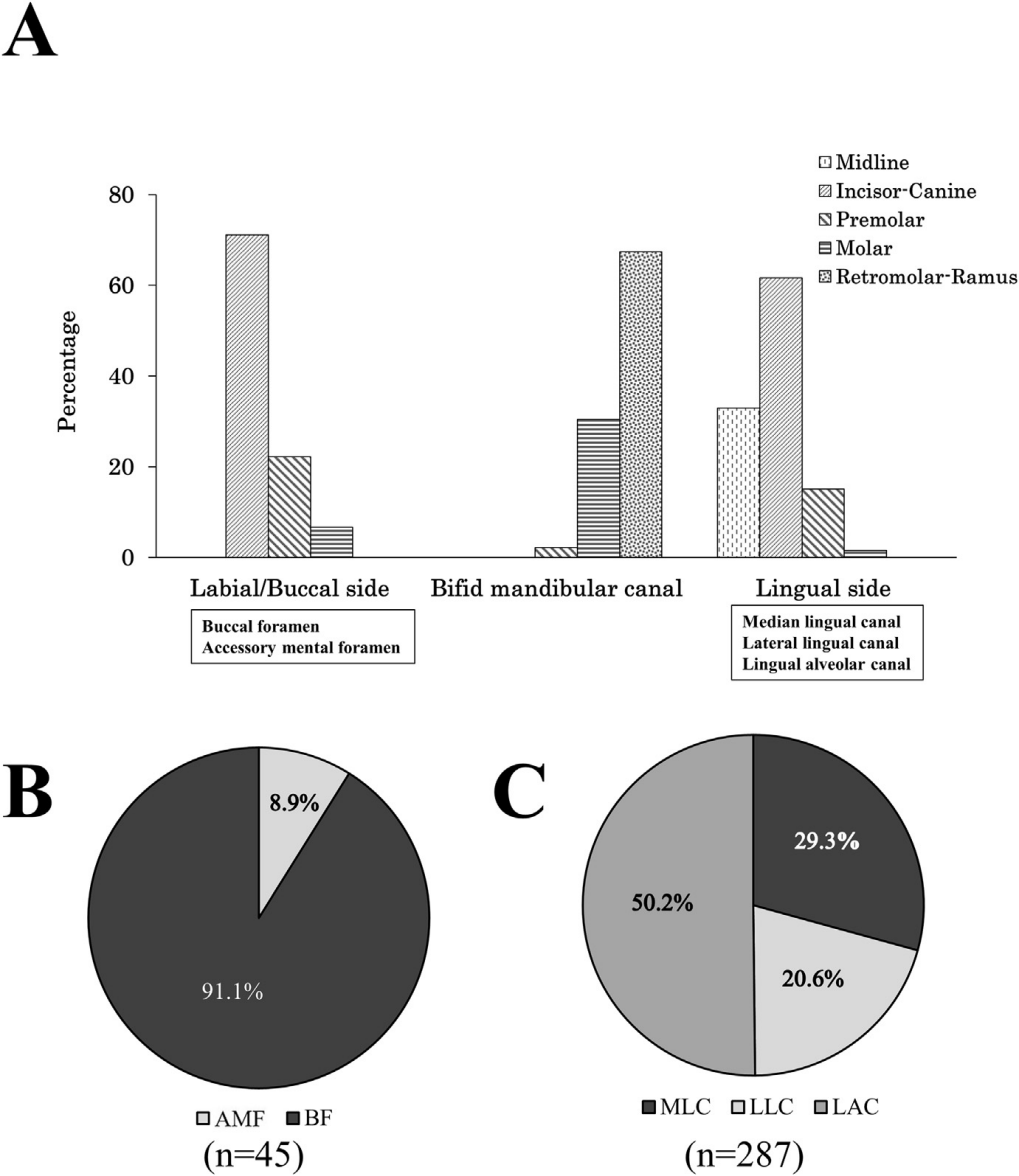
The distribution of neurovascular canals and foramens in the mandible. (A) The percentage of neurovascular canals and foramens on each side and region of mandible. (B) The percentage of neurovascular canals and foramens in the labial and buccal side. (C) The percentage of neurovascular canals and foramens in the lingual side.

The BMC was classified into the following five types: (1) retromolar canal: the canal extends from the inferior alveolar canal (IAC) to the retromolar region (Fig. 1A); (2) dental canal: the canal extends to the root apex of the 2^nd^ or 3^rd^ molar (Fig. 1B); (3) forward canal: the canal courses forward anteriorly and then joins the main IAC (i.e., with confluence; Fig. 1C) or does not join it (i.e., without confluence; Fig. 1D); (4) buccolingual canal: the canal arises from the buccal or lingual wall of the main IAC (Fig. 1E); (5) ramus canal: the canal extends from the IAC to the ramus region, which is above the mandibular foramen (Fig. 1F). An accessory mental foramen arises from the main IAC separately from the mental foramen (Fig. 1G). The MLC, LLC, BF, and LAC opened on the buccal or lingual surface of the mandible (Fig. 1H–K) [19, 20, 21]. The BF is not connected to the IAC. The diameter of these mandibular canals and foramens were measured using a high-resolution picture archiving and communication system (SYNAPSE, version 3.2.1 SR-356; Fujifilm Medical Co., Ltd., Tokyo, Japan). The presence region of these canals and foramens were evaluated.

### 2.4 Detection of neurovascular canals and foramens on panoramic images

Using panoramic images, the BMC was assessed, based on CT findings (Fig. 2). Panoramic images were enhanced and inverted by using a photograph-editing program (picasa3.google.com) for edge enhancement of the IAC, and then analyzed [24].

### 2.5 Statistical analysis

The χ^2^ test was used to assess sex differences in the frequency of neurovascular bone canals and foramens. Statistically significant differences in the diameter of the BMC between undetected canals and detected canals were determined using the Student's *t*-tests. One-way analysis of variance with *post hoc* Tukey's multiple comparison test were used to compare the average diameters of MLC, LLC, BF, and LAC.

Values of *P* < 0.01 were statistically significant. All statistical analyses were performed using EZR (Saitama Medical Center, Jichi Medical University, Shimotsuke, Japan), which is a graphical user interface for R (version 2.13.0, R Foundation for Statistical Computing, Vienna, Austria) [25].

## 3 Results

### 3.1 Study participants

The patients' characteristics are summarized in Table 1. The study involved 104 sides of mandibles from 58 patients. Their mean age was 37.2 ± 10.7 years (range, 18–65 years). There were 20 (34.5%) men and 38 (65.5%) women. There were 53 (51.0%) right side mandibles and 51 (49.0%) left side mandibles.

**Table 1:** Characteristics of the patients and neurovascular canals and foramens.

Variable	Value	
Age, year (mean ± SD (range))	37.2 ± 10.7 (18–65)	
Sex (frequency (%))		
Men	20 (34.5)	
Women	38 (65.5)	
Site of tooth (frequency (%))		
Right	53 (51.0)	
Left	51 (49.0)	
Mandibular neurovascular canal (frequency (%))	In all patients	In all sites
Bifid mandibular canal	35/58 (60.3)	36/104 (34.6)
Accessory mental foramen	4/58 (6.9)	4/104 (3.8)
Median lingual canal	55/56 (98.2)	55/56 (98.2)
Lateral lingual canal	44/58 (75.9)	55/104 (52.9)
Buccal foramen	25/58 (43.1)	32/104 (30.8)
Lingual alveolar canal	57/58 (98.3)	100/104 (96.2)

SD: standard deviation.

### 3.2 The frequency of neurovascular canals and foramens

BMCs in the mandibles existed in 35 (60.3%) of 58 patients and 36 (34.6%) of 104 sides; AMFs, in four (6.9%) of 58 patients and four (3.8%) of 104 sides; MLCs, in 55 (98.2%) of 56 patients; LLCs, in 44 (75.9%) of 58 patients and 55 (52.9%) of 104 sides; BFs, in 25 (43.1%) of 58 patients and 32 (30.8%) of 104 sides; and LACs in 57 (98.3%) of 58 patients and 100 (96.2%) of 104 sides (Fig. 1 and Table 1). No statistically significant sex differences existed among the various neurovascular canals and foramens (data not shown).

### 3.3 The incidence and diameter of the bifid mandibular canal

Twenty-six (25.0%) of 104 sides exhibited only a single canal, 10 (4.4%) of 104 sides exhibited two canals between the premolar region to the ramus region. No BMC had three canals in this study. This study examined the classification of BMC according to a modified version of Naitoh's classification system (Fig. 1A–F) [7]. Among the 104 sides, 11 (10.6%) sides contained a retromolar canal; one (0.9%) side, a dental canal; 24 (23.1%) sides, a forward canal; five (4.8%) sides, a buccolingual canal; and five (4.8%) sides, a ramus canal (Table 2). The average of diameter of the total BMC was 1.22 ± 0.42 mm; the retromolar canal, 1.23 ± 0.38 mm; the dental canal, 0.51 mm; the forward canal, 1.27 ± 0.45 mm; the buccolingual canal, 1.50 ± 0.32 mm; and the ramus canal, 0.87 ± 0.12 mm. Four (3.9%) sides contained AMFs, which had an average diameter of 0.98 ± 0.18 mm (Table 2).

**Table 2:** Characterization of bifid mandibular canals and accessory mental foramens.

	In all patients (frequency (%))	In all sites (frequency (%))	Diameter (mm) (canals ± SD)
Bifid mandibular canal			
Retromolar	10/58 (17.2)	11/104 (10.6)	1.23 ± 0.38
Dental	1/58 (1.7)	1/104 (0.9)	0.51
Forward	20/58 (34.5)	24/104 (23.1)	1.27 ± 0.45
Buccolingual	0/58 (0)	5/104 (4.8)	1.50 ± 0.32
Ramus	5/58 (8.62)	5/104 (4.8)	0.87 ± 0.12
		AVG	1.22 ± 0.42
Accessory mental foramen	4/58 (3.9)	4/104 (3.9)	0.98 ± 0.18

AVG: average, SD: standard deviation.

### 3.4 The incidence and diameter of the MLC, LLC, BF, and LAC

With regard to the MLC among 56 patients, 29 (52.7%) patients had only a single canal, 24 (43.6%) patients had two canals, and two (3.6%) patients had three canals at the midline of mandible (Table 3). With regard to the LLC among 104 sides, 51 (49.0%) sides contained a single canal and four (3.1%) sides contained two canals on the lingual surface of mandible, excluding the ramus regions (Table 3). No LLC had three canals in this study.

**Table 3:** Frequency, location and diameters of the mandibles with median lingual canals (MLC), lateral lingual canal (LLC), buccal foramen (BF) and lateral alveolar canal (LAC).

Canal	MLC	LLC	BF	LAC
Number/frequency (%)				
0 Canal	1/56 (1.8)	49/104 (47.1)	72/104 (69.2)	4/104 (3.9)
1 Canal	29/56 (52.7)	51/104 (49.0)	25/104 (24.0)	59/104 (56.7)
2 Canals	24/56 (43.6)	4/104 (3.1)	5/104 (4.8)	39/104 (37.5)
3 Canals	2/56 (3.6)	0/104 (0)	2/104 (1.9)	2/104 (1.9)
Location				
Midline	56/56 (100)	17/59 (28.8)	32/41 (78.0)	1/144 (0.7)
Incisor-canine	0/56 (0)	38/59 (64.4)	6/42 (14.6)	142/144 (98.6)
Molar	0/56 (0)	4/59 (6.8)	3/42 (7.3)	1/144 (0.7)
Ramus	0/56 (0)	0/59 (0)	0/42 (0)	0/144 (0)
AVG (canals ± SD)	1.48 ± 0.60	0.57 ± 0.57	0.39 ± 0.67	1.38 ± 0.59
Diameter (mm ± SD)	1.05 ± 0.59^a^	0.81 ± 0.41	0.79 ± 0.32	1.00 ± 0.17^a^

AVG: average, SD: standard deviation.

a Significant difference between the MLC and the LLC or BF (*P* > 0.01) and between the LAC and the LLC or BF (*P* > 0.01).

With regard to the BF among 104 sides, 25 (24.0%) sides had a single canal, five (4.8%) sides had two canals, and two (1.9%) sides had three canals on the buccal side of mandible from the incisor to the molar regions (Table 3). With regard to the LAC among 104 sides, 59 (56.7%) sides had a single canal; 39 (37.5%) sides had two canals; and two (1.92%) sides had three canals at the alveolar region of the mandible. Most LACs were in the incisor-canine region (Table 3). Furthermore, for the LAC, four (2.8%) of 144 canals were between the two medial incisors in the mandibles, 93 (64.6%) canals were between the medial and lateral incisor, 16 (11.1%) canals were between the lateral incisor and canine, 30 (20.8%) canals were between the canine and first premolar, and one canal (0.7%) was between the first and second premolars.

The average number of MLCs was 1.48 ± 0.60, LLCs was 0.57 ± 0.57, BFs was 0.39 ± 0.67, and LACs was 1.38 ± 0.59. The average diameter was 1.05 ± 0.59 mm for the MLC, 0.81 ± 0.41 mm for the LLC, 0.79 ± 0.32 mm for the BF, and 1.00 ± 0.17 mm for the LAC (Table 3). There was a significant difference between the MLC and the LLC or BF (*P* > 0.01) and between the LAC and the LLC or BF (*P* > 0.01) (Table 3).

### 3.5 Confirmation of the neurovascular canals and foramens in the panoramic image

Using edge-enhanced inverted images improved image assessment, compared to using original images in endodontics [24, 26]. We were well able to evaluate the outline of the IAC by using this image processing technique. The edge-contrasted inverted panoramic images revealed the BMC in 10 (21.7%) of 46 canals and the AMF in one (25%) of four canals. However, most of these two canals and the other canals and foramens were not clearly visible (Fig. 2). Because of the disordered shadow, it was difficult to identify them in the anterior region, from the incisor to the canine. Furthermore, in many images, the shading failure of the inner surface of the ramus made it difficult to find the neurovascular canals in this region (Fig. 2A and B). In the panoramic images, the mean diameter of the BMC was significantly different between the detected group (1.69 ± 0.20 mm) and the not detected group (1.10 ± 0.38 mm) (*P* < 0.01) (Fig. 2C). Three types of detected canals were the branching type (Fig. 2D), the hypertrophic type (Fig. 2E), and the bifurcation type (Fig. 2F).

### 3.6 The distribution of neurovascular canals and foramen in the mandible

To map the distribution of the canals and foramens in the hemimandible, we divided the mandible into the labial/buccal side, the lingual side, and the bifid mandibular canal. We further subdivided it into the midline, incisor-canine, premolar, molar, and retromolar-ramus regions.

The number of canals and foramens decreased from the anterior region (midline-incisor) to the molar-ramus region on the buccal side and the lingual side. Most BMC were found from the retromolar to the ramus region (Fig. 3A). There were substantially more canals and foramens on the lingual side than on the labial/buccal side (Fig. 3B and C). On the labial/buccal side, the branching rate was 8.9% for the AMF and 91.1% for the BF (Fig. 3B). On the lingual side, the branching rate was 29.3% for the MLC, 20.6% for the LLC, and 50.2% for the LAC (Fig. 3C).

The distribution and occurrence rate for each type of neurovascular canal and foramen appear to differ. Taking into account only the total number of canals on the lingual side, the median area seems as low as 29.3%. However, the MLC has a frequency of 98.2% (Table 1).

## 4 Discussion

This study determined the topographical distribution and diameter of all types of neurovascular canals and foramens in the mandibles of the same patient. Edge-contrast inverted panoramic images more frequently revealed the BMC and AMF. From these detected groups, this study was provided the three characteristic findings of BMC.

The BMC and AMF contain the mandibular nerve, artery, and vein. There rate of BMC has ranged widely for each canal type, based on Naitoh's classification system, in samples used in previous studies: the range was 29.8%–52.5% for the retromolar canal; 4.5%–17.1% for the dental canal; 38.7%–59.6% for the forward canal; and 1.8%–5.4% for the buccolingual canal [1, 8, 15]. The findings in this study were similar to those of previous reports in that the retromolar canal and forward canal constituted most canals. Muinelo-Lorenzo et al. [1] reported that the mean diameter for the total BMC was 1.6 ± 0.7 mm; for the retromolar canal, 1.6 ± 0.7 mm; for the forward canal, 1.5 ± 0.4 mm; for the dental canal, 2.0 ± 1.1 mm; and for the buccolingual canal, 1.5 ± 0.7 mm; the diameters of the BMC were narrower in the current study (Table 2). Toh et al. [27] reported that the diameter of the AMF was 0.74–0.89 mm.

The sublingual and the submental artery and vein enter the lingual cortical plate of the mandible through the MLC and LLC [19, 28]. Wang et al. [19] reported that the detection frequency of the MLC was 97.0% and the LLC was 99.0%, which was similar to our results (98.2%) (Table 1). The number of branches of the MLC was 2.01 ± 0.83 and the LLC was 2.95 ± 1.21, which was greater than in our study (Table 3) [19]. The diameter of the MLC and LLC was 0.61 ± 0.33 mm and 0.58 ± 0.30 mm, respectively, based on CBCT [19]. This finding was smaller than in our study (Table 3). Wang et al. reported that most (74.6%) LLCs were in the incisor region, 19.7% of LLCs were in the premolar region, and 5.7% of LLC were in the molar region. However, the current study indicated that most (64.4%) LLCs were in the premolar region (Table 3) [19].

Previous reports suggest that the submental, lower lip, and buccal arteries, and direct branches of the facial artery enter the buccal cortical bone of the mandible through the BF, and that some BFs were continuous with the incisive canal or the mandibular canal [20]. This finding suggests that the BF contains blood vessels and nerve fibers. Naitoh et al. reported that 49.4% of BFs in the mandible are in the median and incisor region; 33.8%, in the premolar region; and 16.9%, in the posterior to the mental foramen [20]. The BF had a high frequency in the incisor region in the current study (Table 3).

The sublingual artery and vein enter the lingual alveolar region of the mandible through the LAC [28]. Murlimanju et al. [19] reported LACs in 96% of human adult dry mandibles, which was similar to our findings (Table 1), and 1.9% of LACs were between the two medial incisors; 50.7%, between the medial and lateral incisor; 10.4%, between the lateral incisor and canine; and 8.9%, between the canine and first molar. In our study, the LAC were more frequently between the medial and lateral incisors and less frequently between the lateral incisors and canines.

Previous investigators have reported that the prevalence rate of BMCs was 0.08%–8.3%, based on panoramic images [1]. Some researchers have reported that the rate of confirmation of BMCs on panoramic images, compared with CBCT, was approximately 40% [1, 8]. Bogdán et al. [29] found that 19.6% of dry mandibles in their study contained BMCs; however, 0.2% of total cases were visible on panoramic images. The use of edge-contrasted inverted digital x-ray improved image assessment in endodontics; therefore, in this study this image processing technique was used for the panoramic images [24, 26]. In fact, the IAC was visualized more clearly by using this tool. However, only 21.7% of BMCs were clearly confirmed in this study. Because of soft tissues, the submandibular fossae and inner surface of the ramus may produce ghost shadows and interfere with the visualization of neurovascular canals; thus, most BMCs were not detected (Fig. 2A and B). Muinelo-Lorenzo et al. [1] reported significantly thicker BMCs on panoramic images, compared to those that could not be detected, which was similar to our findings. Hence, these data suggest that thickness is associated with what can be detected by panoramic images. Furthermore, this study found that emphasizing the contour of the IAC using image processing is useful for finding canals and the characteristic three findings of the BMC in panoramic images. The AMF can rarely be confirmed with panoramic images because the diameter of the AMF is generally <1 mm [18]. This study indicated that the average AMF diameter is <1 mm (0.98 mm). The AMF was detected on panoramic images in only one of four sides with CT images. The BF diameter was <1 mm (0.79 mm), similar to the AMF; therefore, this study could not detect the BF clearly on panoramic images. The detection of the MLC, LLC, BF, and LAC was difficult on panoramic images because they were overlapped with the teeth, the trabecular and lingual cortical bone, the opposite mandible, and cervical vertebrae on panoramic images [20].

All canals and foramens in the mandible are commonly exposed to local anesthetic procedures, mandibular cystectomy, and mandibular tumor resection. Most BMCs are in the molar and ramus regions. Oral surgeons need to carefully avoid injuring the BMC, which is in the ramus region, during extraction of impacted third molars and orthognathic surgeries. Surgeries that are performed in the molar region are third molar extraction, flap operation, and angle-splitting osteotomy. Surgeons need to identify the AMF when performing surgical procedures in the premolar region (e.g., extraction, flap operation, dental implant operation, anterior segmental osteotomy). The MLC and BF need to be identified during operations for extraction, flap operation, dental implant operation, and genioplasty in the incisor region. The LLC needs to be approached with caution during extractions, dental implant operations, and torus removal in the premolar region. The LAC was in the incisor region. It may need to be approached with caution during extraction and dental implant operations.

Intraoral bleeding is a very serious situation when it obstructs the airway [6]. Previous reports [19] suggest that small blood vessels with a diameter <1 mm are rarely problematic when resecting these blood vessels. However, when a larger blood vessel is injured, unexpected bleeding causes a serious incident. By using ultrasound Doppler imaging, Lustig et al. [30] demonstrated that an arterial diameter of 0.18–1.8 mm causes a blood flow of 0.7–3.7 mL/s, which explains the profuse hemorrhaging on injury.

This study may contribute to the ability to predict the location of neurovascular canals and foramens, to accurately identify them, and to avoid complications during oral surgical techniques. Oral surgeons should carefully avoid complications because canals that are detectable on panoramic images are often thick.

## Declarations

### Author contribution statement

Shigehiro Abe: Conceived and designed the experiments; Performed the experiments; Analyzed and interpreted the data; Wrote the paper.

Ayumi Moro: Performed the experiments; Analyzed and interpreted the data; Wrote the paper.

Naoko Yokomizo: Performed the experiments.

Yutaka Kobayashi: Performed the experiments; Contributed reagents, materials, analysis tools or data.

Takashi Ono, Toshiaki Takeda: Performed the experiments; Contributed reagents, materials, analysis tools or data.

### Funding statement

This research did not receive any specific grant from funding agencies in the public, commercial, or not-for-profit sectors.

### Competing interest statement

The authors declare no conflict of interest.

### Additional information

No additional information is available for this paper.
