# Corrigendum to “Topographical distribution of neurovascular canals and foramens in the mandible: avoiding complications resulting from their injury during oral surgical procedures” [Heliyon 4 (9) (September 2018) e00812]

**DOI:** 10.1016/j.heliyon.2018.e00946

**Published:** 2018-12-06

**Authors:** Ayumi Moro, Shigehiro Abe, Naoko Yokomizo, Yutaka Kobayashi, Takashi Ono, Toshiaki Takeda

**Affiliations:** a Department of Dentistry and Oral Surgery, Tokyo Metropolitan Hiroo Hospital, 2-34-10 Ebisu, Shibuya-ku, Tokyo, 150-0013, Japan; b Department of Radiology, Tokyo Metropolitan Hiroo Hospital, 2-34-10 Ebisu, Shibuya-ku, Tokyo, 150-0013, Japan

In the original published version of this article, an error was present in two of the tables. There were mistakes in the numbers in Table 2 and 3. The location of premolar was missing in Table 3. In Table 2, the frequency of the buccolingual canal in all patients (%) was 0/58 (0) (the correct number is 5/58 (8.6)), and the frequency of the accessory mental foramen was 4/58 (3.9) (correct number is 4/58 (6.9)). These errors were typographical errors. The total number of medial lingual canals (MLCs) was 56 (correct number is 55), and the total number of buccal foramens (BFs) was 42 (correct number is 41) in Table 3. These errors were also typographical errors. However, the frequencies (%) in Table 3 were not incorrect. Although the lateral lingual canal (LLC) and the BF did not exist in the midline region, these data were missing in Table 3. Furthermore, because there was no mention of the premolar region, these errors confused readers. However, there were no mistakes in the data provided in the text of this article. The authors apologize for these mistakes. A corrected Table 2 and Table 3 are displayed below with the changed values in bold. This correction does not, in any way, compromise the findings of the study, either in terms of the methodology, results, or interpretations drawn from the data therein.

**Table 2**. Characterization of bifid mandibular canals and accessory mental foramens.

**Table:** 

	in all patients (frequency (%))	in all sites (frequency (%))	Diameter (mm) (canals ± SD)
Bifid mandibular canal			
Retromolar	10/58 (17.2)	11/104 (10.6)	1.23 ± 0.38
Dental	1/58 (1.7)	1/104 (0.9)	0.51
Forward	20/58 (34.5)	24/104 (23.1)	1.27 ± 0.45
Buccolingual	**5/58 (8.6)**	5/104 (4.8)	1.50 ± 0.32
Ramus	5/58 (8.62)	5/104 (4.8)	0.87 ± 0.12
		AVG	1.22 ± 0.42
Accessory mental foramen	4/58 (**6.9**)	4/104 (3.9)	0.98 ± 0.18

AVG: average, SD: standard deviation.

**Table 3**. Frequency, location and diameters of the mandibles with median lingual canals (MLC), lateral lingual canal (LLC), buccal foramen (BF) and lateral alveolar canal (LAC).

**Table:** 

Canal	MLC	LLC	BF	LAC
Number/Frequency (%)				
0 canal	1/56 (1.8)	49/104 (47.1)	72/104 (69.2)	4/104 (3.9)
1 canal	29/56 (52.7)	51/104 (49.0)	25/104 (24.0)	**59/104 (56.7)**
2 canals	24/56 (43.6)	4/104 (3.1)	5/104 (4.8)	39/104 (37.5)
3 canals	2/56 (3.6)	0/104 (0)	2/104 (1.9)	2/104 (1.9)
Location				
Midline	**55/55 (100)**	**0/59 (0)**	**0/41 (0)**	**1/144 (0.7)**
Incisor-canine	**0/55 (0)**	**17/59 (28.8)**	**32/41 (78.0)**	**142/144 (98.6)**
**Premolar**	**0/55 (0)**	**38/59 (64.4)**	**6/41 (14.6)**	**1/144 (0.7)**
Molar	**0/55 (0)**	**4/59 (6.8)**	**3/41 (7.3)**	**0/144 (0)**
Ramus	**0/55 (0)**	**0/59 (0)**	**0/41 (0)**	**0/144 (0)**
AVG (canals ± SD)	1.48 ± 0.60	0.57 ± 0.57	0.39 ± 0.67	1.38 ± 0.59
Diameter (mm ± SD)	1.05 ± 0.59*	0.81 ± 0.41	0.79 ± 0.32	1.00 ± 0.17*

AVG: average, SD: standard deviation.

*: Significant difference between the MLC and the LLC or BF (*P* > 0.01) and between the LAC and the LLC or BF (*P* > 0.01).

